# Matryoshka-Type Liposomes Offer the Improved Delivery of Temoporfin to Tumor Spheroids

**DOI:** 10.3390/cancers11091366

**Published:** 2019-09-13

**Authors:** Ilya Yakavets, Marie Millard, Laureline Lamy, Aurelie Francois, Dietrich Scheglmann, Arno Wiehe, Henri-Pierre Lassalle, Vladimir Zorin, Lina Bezdetnaya

**Affiliations:** 1 Centre de Recherche en Automatique de Nancy, Centre National de la Recherche Scientifique UMR 7039, Université de Lorraine, Campus Sciences, Boulevard des Aiguillette, 54506 Vandoeuvre-lès-Nancy, France; 2 Research Department, Institut de Cancérologie de Lorraine, 6 avenue de Bourgogne, 54519 Vandoeuvre-lès-Nancy, France; 3 Laboratory of Biophysics and Biotechnology, Belarusian State University, 4 Nezavisimosti Avenue, 220030 Minsk, Belarus; 4 Biolitec research GmbH, Otto-Schott-Strasse 15, 07745 Jena, Germany; 5 International Sakharov Environmental Institute, Belarusian State University, Dauhabrodskaja 23, 220030 Minsk, Belarus

**Keywords:** temoporfin, drug-in-cyclodextrin-in-liposome, hybrid nanoparticles, multicellular tumor spheroids, cyclodextrins, photodynamic therapy article, yet reasonably common within the subject discipline

## Abstract

The balance between the amount of drug delivered to tumor tissue and the homogeneity of its distribution is a challenge in the efficient delivery of photosensitizers (PSs) in photodynamic therapy (PDT) of cancer. To date, many efforts have been made using various nanomaterials to efficiently deliver temoporfin (mTHPC), one of the most potent photosensitizers. The present study aimed to develop double-loaded matryoshka-type hybrid nanoparticles encapsulating mTHPC/cyclodextrin inclusion complexes in mTHPC-loaded liposomes. This system was expected to improve the transport of mTHPC to target tissues and to strengthen its accumulation in the tumor tissue. Double-loaded hybrid nanoparticles (DL-DCL) were prepared, characterized, and tested in 2D and 3D in vitro models and in xenografted mice in vivo. Our studies indicated that DL-DCL provided deep penetration of mTHPC into the multicellular tumor spheroids via cyclodextrin nanoshuttles once the liposomes had been destabilized by serum proteins. Unexpectedly, we observed similar PDT efficiency in xenografted HT29 tumors for liposomal mTHPC formulation (Foslip^®^) and DL-DCL.

## 1. Introduction

Nanomaterials are the cornerstone in the rapidly advancing field of nanotechnology, playing a crucial role in successful drug delivery at diseased sites [1]. To date, many nanoplatforms have been applied for the delivery of temoporfin (5,10,15,20-tetra(m-hydroxyphenyl)chlorin, mTHPC), one of the most promising photosensitizers (PSs) used in the photodynamic therapy (PDT) of solid cancers. Temoporfin has been marketed in the European Union since 2001 under the trade name Foscan^®^ (biolitec pharma Ltd., Jena, Germany), and is indicated for the palliative treatment of head and neck squamous cell carcinoma [2]. Nanodelivery systems were supposed to overcome or improve the major constraints of mTHPC, such as low solubility, unfavorable pharmacokinetic profiles, and side effects (pain upon injection, skin photosensitivity) [3]. However, due to the complexity of drug distribution processes, the use of individual NPs offered neither optimal, leakage-free delivery of mTHPC to the tumor nor the local release of large amounts of mTHPC. At the same time, multifunctional nanomedicines featuring high drug loading capacity, controllable drug release, and real-time self-monitoring are attracting immense interest due to their potential to improve cancer therapy efficacy [4,5,6]. 

**Figure 1:**
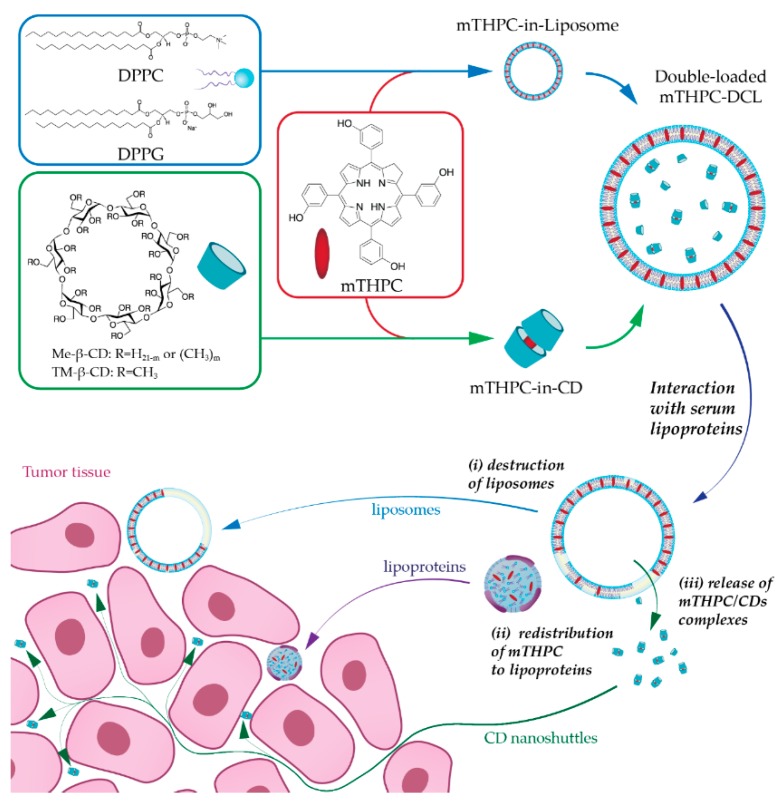
Double-loaded matryoshka-typemTHPC drug-in-cyclodextrin-in-liposomes (mTHPC-DCLs). Schematic illustration of the serum-mediated mTHPC release and penetration into the tumor tissue: (***i***) serum proteins disintegrate liposomal bilayer resulting in (***ii***) redistribution of lipid bilayer components (lipids and mTHPC) to serum lipoproteins (***iii***) as well as the release of water-soluble mTHPC/CD (cyclodextrin) inclusion complexes. Liposomes (blue arrow, Foslip^®^) and serum lipoproteins (purple arrow) interact only with outer layer cells, while CDs (green arrow) can penetrate the tumor tissue and deliver PS.

In the present study, we have suggested combining NPs into one nanoplatform as an advanced alternative strategy for mTHPC delivery. Recently, we reported the encapsulation of mTHPC-cyclodextrin (CD) supramolecular complex into liposomes, namely drug-in-cyclodextrin-in-liposome (DCL), as a prospective nanodelivery system for mTHPC [7]. The upgraded double-loaded mTHPC-DCLs (DL-DCL) contained mTHPC in both lipid and aqueous compartments of lipid vesicles. We hypothesized that such “matryoshka-type” hybrid liposomes would combine the advantages of each delivery system (Figure 1). Liposomes are effective containers for selective mTHPC delivery to target sites [8]. However, liposomes have limited penetration into deep tissue layers [9,10]. Alternatively, mTHPC/CDs inclusion complexes easily penetrate tumor tissue, thereby significantly increasing PS accumulation [11]. However, these supramolecular complexes are prone to dissociation in vivo, once diluted in the bloodstream [12]. Thus, we suggested that the coupling of both delivery systems intp one DL-DCL could restrain the dissociation of drug–mTHPC complexes, avoid rapid drug release, and favorably alter PS penetration into tumor tissues.

In the present work, we prepared and characterized DL-DCLs and tested these complexes in 2D and 3D tumor cell cultures. We focused on the study of double-loaded mTHPC-DCLs in 3D multicellular tumor spheroids in terms of PS penetration and accumulation. Finally, we conducted a preliminary study on double-loaded mTHPC-DCL efficacy in vivo in tumor-xenografted mice.

## 2. Results

### 2.1. Characterization

**Figure 2:**
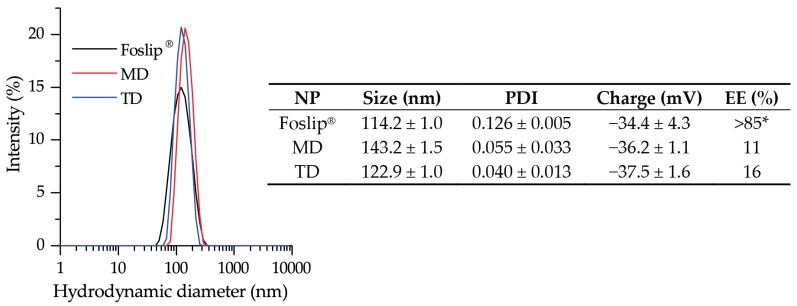
The hydrodynamic size of Foslip^®^ (black) and DL-DCLs: MD (red) and TD (blue). Insert shows the physico-chemical characteristics of the NPs of hydrodynamic diameter (nm), polydispersity index (PDI), Z-potential (mV), and encapsulation efficiency (EE,%). *—taken from Reference [13]. Abbreviations MD and TD stand for DL-DCL with encapsulated mTHPC-Methyl-β-CD or mTHPC-Trimethyl-β-CD complexes, respectively.

We prepared DL-DCLs with mTHPC encapsulated in the lipid membrane as well as in the aqueous core in the soluble form of inclusion complexes with β-CDs. Abbreviations MD and TD stand for DL-DCL with encapsulated mTHPC-Methyl-β-CD or mTHPC-Trimethyl-β-CD complexes, respectively. The hydrodynamic size, polydispersity index (PDI), and Zeta-potential of MD and TD were measured by dynamic light scattering. Both DL-DCLs had a narrow size distribution with a mean hydrodynamic diameter of 143.2 ± 1.5 nm for MD (PDI = 0.055 ± 0.033) and 122.9 ± 1.1 nm for TD (PDI = 0.040 ± 0.013) (Figure 2). The surface charge of MD and TD was negative, with Zeta potentials of −36.2 ± 4.3 mV and −37.5 ± 1.6 mV, respectively. It is worth noting that DL-DCLs have high colloidal stability (>3 months) (data not shown). The encapsulation efficiencies (EE) of mTHPC in MD and TD were estimated as 11% and 16%. As control NPs, we used a conventional liposomal mTHPC formulation (Foslip^®^) with a hydrodynamic size of 114.2 ± 1.0 nm (PDI = 0.110 ± 0.015) and a Zeta potential of −34.4 ± 4.3 mV.

**Figure 3:**
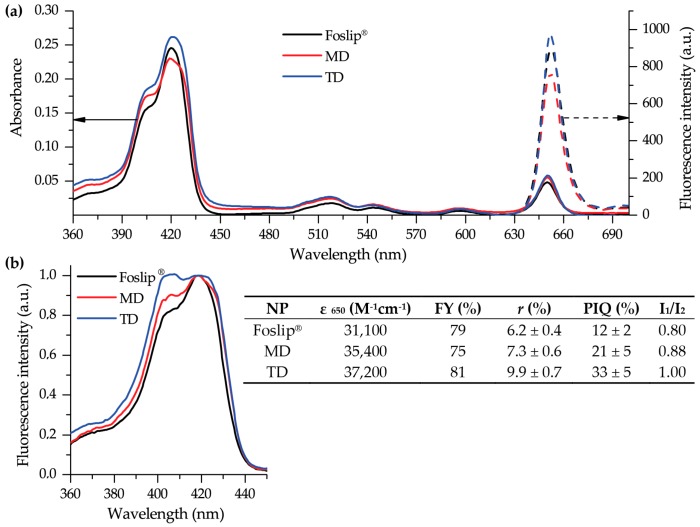
Spectral characteristics of Foslip^®^ (black), MD (red), and TD (blue) in PBS. (**a**) Absorbance (solid line) and fluorescence emission (dotted line) spectra (λ_ex_ = 420 nm); (**b**) normalized fluorescence excitation spectra (λ_em_ = 652 nm). mTHPC concentration was 1.5 μM. Insert shows the main spectral characteristics of NPs, such as extinction coefficient at 650 nm (ε_650_); FY—fluorescence yield, relative to the fluorescence of mTHPC in ethanol; *r*—the degree of fluorescence anisotropy; PIQ—the degree of photoinduced quenching; I_1_/I_2_—the ratio of Soret band components (I_1_ and I_2_ were measured under excitation at 407 nm and 418 nm, respectively, and emission at 652 nm).

Spectral characteristics of mTHPC-based nanoformulations in PBS are presented in Figure 3. All mTHPC formulations exhibited narrow spectral bands, indicating the monomeric state of mTHPC. The absorption spectra of Foslip^®^, MD, and TD in PBS were characterized by a Soret band (maximum at 416 nm) and four Q-bands with prominent peaks at 650 nm (Figure 3a). The extinction coefficients of mTHPC in DL-DCLs were slightly higher (35,400 M^−1^cm^−1^ and 37,200 M^−1^cm^−1^) than that of Foslip^®^ (31,100 M^−1^cm^−1^). The mTHPC fluorescence was emitted at 652 nm for all NPs. The relative fluorescence quantum yield (FY) of mTHPC encapsulated in DL-DCLs was comparable with Foslip^®^ and was only 20% lower than a standard mTHPC ethanol solution. Figure 3b exhibits the excitation fluorescence spectra of mTHPC in various NPs in the Soret band region, which is considered to be sensitive to the binding of mTHPC with β-CDs [14]. The relative fluorescence in a short wavelength shoulder was significantly increased in DL-DCLs compared with Foslip^®^ due to the formation of inclusion complexes between mTHPC and β-CD derivatives. To assess the changes in the shape of spectral band, we calculated I_1_/I_2_, where I_1_ and I_2_ stand for the fluorescence intensities at 407 nm and 418 nm excitation, respectively. The calculated I_1_/I_2_ ratios for mTHPC in MD and TD were 0.88 and 1.00, while that of Foslip^®^ was 0.80 (Figure 3b, insert). The microenvironment of mTHPC in NPs could also be characterized using fluorescence parameters such as fluorescence anisotropy and photoinduced fluorescence quenching (PIQ). Consistent with our previous report [13], the fluorescence anisotropy (*r*) of mTHPC in Foslip^®^ was 6.2 ± 0.4%, and the PIQ was 12 ± 2 % (Figure 3b, insert). In DL-DCL, both fluorescence anisotropy and PIQ values were increased, providing r = 7.3 ± 0.6% and PIQ = 21 ± 5% for MD, and r = 9.9 ± 0.7% and PIQ = 33 ± 5% for TD. Based on these values, we assessed that about 70% of mTHPC in the DL-DCLs was attached to the lipid bilayer, while 30% of PS was bound to CDs in the inner aqueous cores of the lipid vesicles.

### 2.2. Two-Dimensional (2D) Monolayer Cell Culture

**Figure 4:**
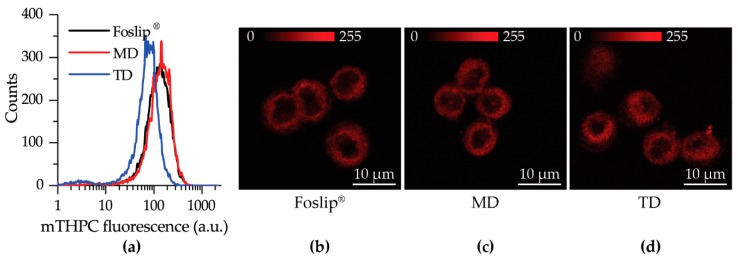
(**a**) Flow cytometry histograms of HT29 monolayer cells treated with Foslip^®^ (black), MD, (red) and TD (blue) for 24 h; (**b**–**d**) Typical confocal images of mTHPC fluorescence in HT29 monolayer cells at 3 h post-incubation with (**b**) Foslip^®^, (**c**) MD, and (**d**) TD. Scale bar: 10 μm. The concentration of mTHPC was 1.5 μM. Serum concentration was 2%.

The cellular uptake of mTHPC delivered by Foslip^®^ or DL-DCLs was analyzed by flow cytometry. Flow cytometry histograms of HT29 human colon adenocarcinoma monolayer cells treated for 24 h with Foslip^®^, MD, and TD (1.5 μM) are presented in Figure 4a. All profiles had a narrow homogeneous distribution. The accumulation of mTHPC in HT29 monolayer was slightly lower for TD compared with MD and Foslip^®^. The estimated mean cellular fluorescent intensities for Foslip^®^ and MD were 140 ± 14 a.u. and 149 ± 21 a.u. (*p* > 0.05), respectively, while that for TD was significantly lower (80 ± 9 a.u.; *p* < 0.05).

Intracellular localization of mTHPC in HT29 monolayer cells was evaluated using confocal microscopy after 3 h of incubation (Figure 4b–d). No remarkable difference in intracellular mTHPC localization between Foslip^®^ and DL-DCLs was observed. All NPs exhibited a similar mTHPC fluorescence pattern in cells, characterized by diffuse fluorescence in the cytoplasm outside both the nucleus and outer plasma membrane. Similar localization patterns were observed in human pharynx squamous cell carcinoma (FaDu) (Figure S1).

### 2.3. Three-Dimensional (3D) Multicellular Tumor Spheroids

#### 2.3.1. Accumulation and Distribution

**Figure 5:**
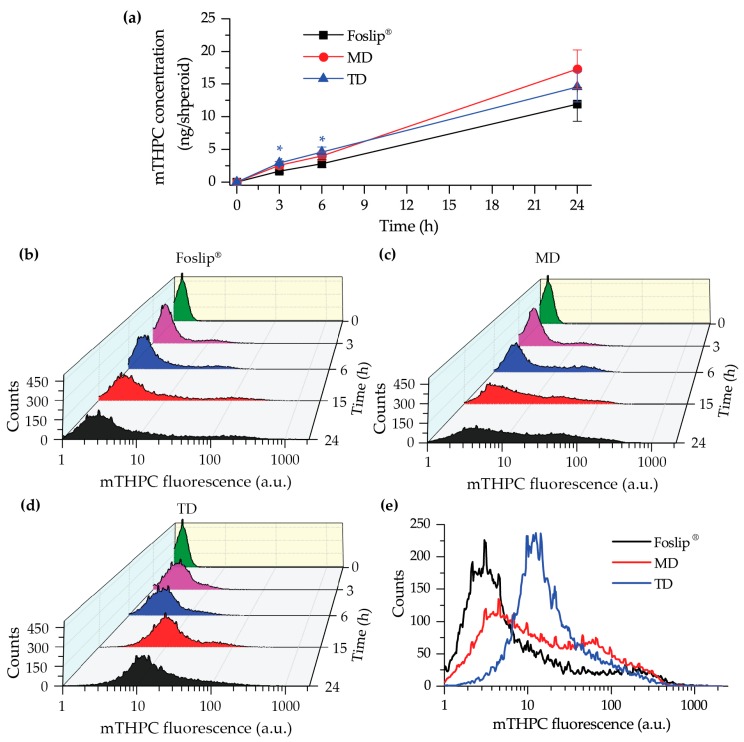
(**a**) Kinetics of mTHPC uptake in HT29 spheroids after incubation with Foslip^®^ (black), MD (red), and TD (blue) using chemical extraction in absolute ethanol. The data are presented as mean ± standard deviation. * *p* < 0.05 compared to Foslip^®^; (**b**–**e**) Typical flow cytometry histograms of trypsinized spheroids treated with (**b**) Foslip^®^, (**c**) MD, and (**d**) TD at 3, 6 , 15, and 24 h post-incubation. (**e**) Typical flow cytometry histograms of HT29 spheroids treated with Foslip^®^ (black), MD (red), and TD (blue) for 24 h. mTHPC concentration was 4.5 μM. Serum concentration was 2%.

We performed chemical extraction of mTHPC from HT29 spheroids after 3, 6, and 24 h incubation with Foslip^®^ or DL-DCLs (4.5 µM) (Figure 5a). Accumulation of mTHPC in spheroids for all NPs increased continuously over 24 h. At short incubation times (3 and 6 h), mTHPC accumulation was slightly but significantly higher in TD-treated spheroids compared to those treated with Foslip^®^ (*p* > 0.05). At 24 h incubation, the highest amount of mTHPC was observed in MD-treated spheroids (17.3 ± 2.9 ng/spheroid), although it was not significant compared with TD and Foslip^®^ (14.6 ± 2.3 and 11.9 ± 2.6 ng/spheroid, respectively; *p* > 0.05). 

Afterwards, we assessed the accumulation of mTHPC in individual cells in spheroids using flow cytometry analysis (Figure 5b–e and Figures S2 and S3). Figure 5b–d displays the kinetics of mTHPC uptake in HT29 spheroids treated with various nanoformulations. We observed that Foslip^®^ continuously accumulated from 3 h incubation, but only in a small fraction of cells, thus resulting in a strongly heterogeneous distribution of mTHPC across the spheroids. On the other hand, spheroids treated with MD demonstrated a more homogeneous distribution, especially visible after 24 h of incubation (Figure 5e). Figure 5e displays a typical distribution of mTHPC in HT29 spheroids treated with nanoformulations, while the histograms from independent experiments are presented in Figure S2. Finally, the fluorescence in TD treated spheroids represented one narrow peak in the histogram irrespective of incubation time, indicating an almost homogeneous mTHPC distribution across spheroids. FaDu spheroids were assessed after 24 h incubation with nanoformulations (Figure S3). Foslip^®^ and TD provided distribution profiles in FaDu spheroids similar to those in HT29 spheroids, while the MD distribution was more heterogenous compared with that in HT29. 

#### 2.3.2. Penetration

**Figure 6:**
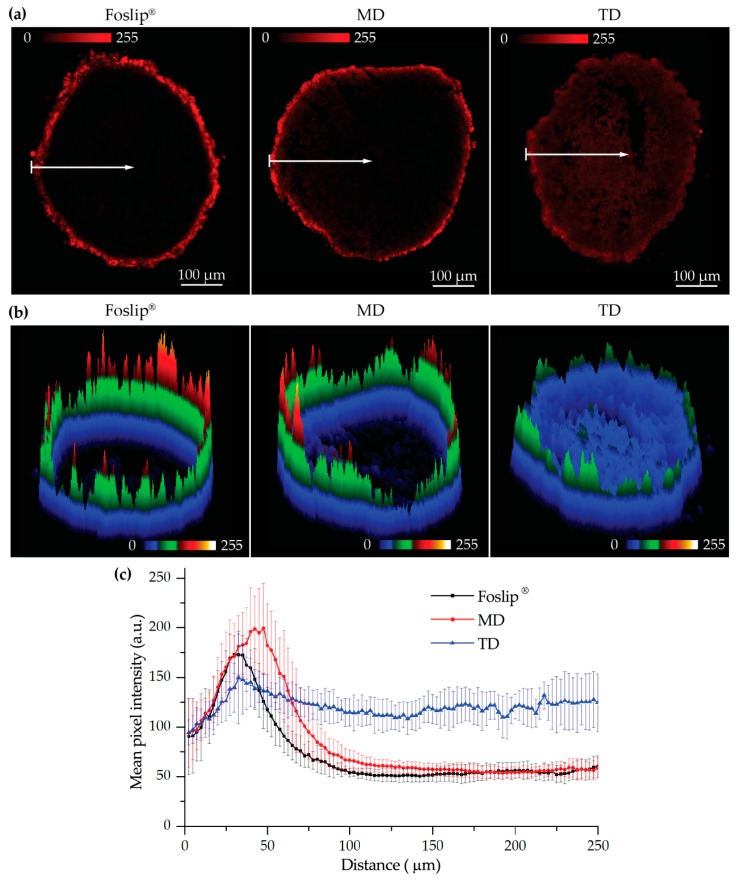
(**a**) Typical fluorescence images and corresponding (**b**) surface plots of mTHPC fluorescence in HT29 spheroids after 24 h incubation with various mTHPC formulations (Foslip^®^, MD, and TD); (**c**) penetration profiles of Foslip^®^ (black), MD (red), and TD (blue) in HT29 spheroids after 24 h. The data are presented as mean ± standard deviation obtained from *n* = 7 spheroids. mTHPC concentration was 4.5 μM. Serum concentration was 2%. mTHPC fluorescence is displayed in red color (2D images) and in pseudo-colors (3D surface plots).

To confirm the results of the flow cytometry-based distribution, we analyzed cryosections of spheroids treated with NPs for 24 h using laser scanning confocal microscopy (Figure 6a). For a better comparison of mTHPC distribution in spheroids, the images were completed with surface plots of fluorescence patterns (Figure 6b). As seen in Figure 6a,b, Foslip^®^ and MD displayed strong mTHPC fluorescence only on the periphery of the spheroids, while for TD, mTHPC fluorescence was observed across the whole spheroid, demonstrating complete PS penetration. Similar patterns of mTHPC distribution were observed in FaDu spheroids treated with Foslip^®^ and TD (Figure S4). The comparison of linear profiles demonstrated a slightly deeper penetration of MD in HT29 spheroids compared with Folsip^®^. However, the mTHPC fluorescence signal at 100 µm from the periphery of the spheroids was undetectable for both formulations. At the same time, the fluorescence of mTHPC delivered by TD was almost constant throughout the whole spheroid depth.

#### 2.3.3. Serum-Induced Release of CDs

**Figure 7:**
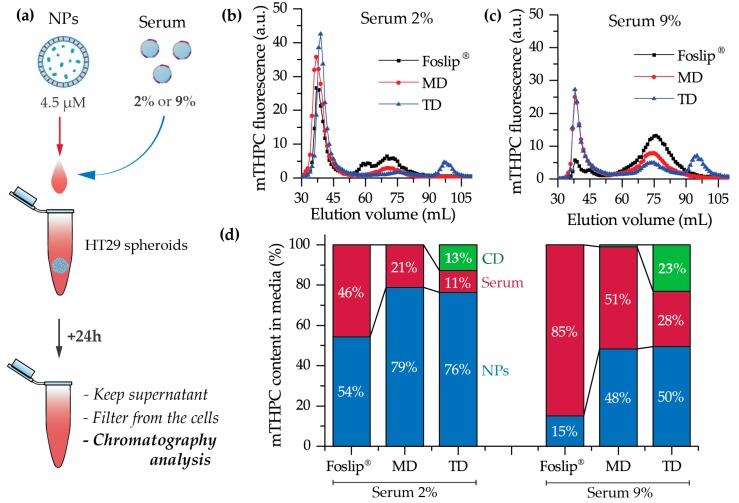
(**a**) Scheme of the experiment; (**b**,**c**) chromatography histograms of culture medium after 24 h incubation of HT29 spheroids with mTHPC (4.5 μM) in nanoformulations in the presence of (**b**) 2% and (**c**) 9% of serum; (**d**) the percentage of mTHPC bound with NPs (blue) or serum proteins (red) and in complexes with CDs (green).

In order to confirm the release of CDs upon serum-induced destabilization of the liposomal vesicles, we analyzed mTHPC fluorescence intensities in the medium supplemented with serum at different concentrations (Figure 7a). After 24 h incubation of spheroids with NPs, the collected supernatant was centrifugated, filtered, and analyzed by gel exclusion chromatography. Figure 7b,c represents the distribution of mTHPC between eluted fractions. Based on the calibration experiment (data not shown), the elution range of liposomes and DL-DCLs was 30–45 mL, serum protein fractions were eluted at 50–90 mL, and mTHPC-CD complexes, as the smallest in size, were eluted only at 90–105 mL. The estimated percentage of mTHPC bound to each fraction is displayed in Figure 7d. According to these data, mTHPC was efficiently released from Foslip^®^ and 50% of PS was bound to serum proteins in the medium enriched with 2% of serum. When the percentage of serum was increased up to 9%, only 15% of mTHPC was detected in liposomes. On the other hand, both DL-DCLs contained higher mTHPC concentrations after 24 h incubation with spheroids compared to Foslip^®^. The remaining fraction of mTHPC in MD and TD for 2% of serum was 79% and 76%, respectively, and 48% and 50% for 9% serum. Most importantly, we directly detected the presence of mTHPC-CD complexes in the medium after incubation of the spheroids with TD, and the fraction of mTHPC-CD complexes increased from 13% to 23% when the serum content in the medium increased from 2% to 9%. Alternatively, in the case of MD, the released mTHPC was bound to serum proteins only.

### 2.4. PDT

Considering that better penetration and distribution of mTHPC delivered by DL-DCLs could improve PDT efficiency, we assessed the viability of HT29 spheroid cells using a propidium iodide fluorescent probe. Spheroids, treated with NPs for 24 h were irradiated at 40 J/cm² (90 mW/cm^2^), trypsinated 6 h later, and analyzed by flow cytometry. Cell viability in control (no-drug, no-light) and (no-light) groups was about 82% for all NPs. PDT resulted in a significant decrease in cell viability, yielding about 40% necrotic cells. Surprisingly, photoinduced necrosis was similar for all types of NPs (39 ± 2% vs. 40 ± 4% vs. 41 ± 2% for Foslip^®^, MD, and TD, respectively; *p* > 0.05).

**Figure 8:**
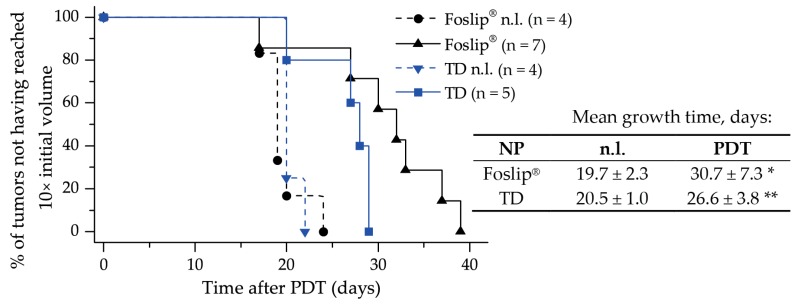
Kaplan–Meier plots of HT29 tumor growth delay and mean tumor growth time (time to reach 10× the initial tumor volume) of control no-light (n.l.) groups and mice after PDT with Foslip^®^ and TD at 24 h of drug-light-interval. * statistically different from Foslip^®^ n.l. control group, *p* < 0.05; ** statistically different from TD n.l. control group, *p* < 0.05.

We further studied PDT tumor response in vivo in a xenografted mice model. Kaplan–Meier plots of tumor response to Foslip^®^- and TD–PDT are presented in Figure 8. The tumor growth delay was about 30.7 days for Foslip^®^ and 26.6 days for TD (*p* > 0.05). For both NPs, the response was significantly different from the control no-light (n.l.) groups (*p* < 0.05).

## 3. Discussion

To date, hybrid NPs, combining several nanomaterials in one, have attracted increasing attention as anti-cancer delivery systems [15]. The careful selection of the combined nanomaterials could result in a considerable synergistic effect overcoming the individual nanostructures’ limitations. In the case of mTHPC, a synergy could be achieved by a coupling of CD-based nanoshuttles and liposomes. mTHPC exhibits an extremely strong affinity to methylated β-CDs (10^6^–10^7^ M^−1^ for Me-β-CD and TM-β-CD, respectively [16]) leading to the unique possibility of altering mTHPC biodistribution via a nanoshuttle mechanism [11,17]. At the same time, liposomal formulations of mTHPC display selective delivery of PS to tumor tissue [8]. CDs could be regarded as an added value to mTHPC-based liposomes, and as such, could increase drug loading capacity, entrapment efficiency, prolong the circulation time of the drug in the bloodstream, reduce toxicity, and finally provide controlled release [18,19]. Thus, we supposed that such a “matryoshka-doll” nanostructure could be a potent delivery system, providing both passive tumor targeting by liposomes and deep penetration of mTHPC/CD complexes into the tumor tissue (Figure 1).

Recently, we optimized the composition of mTHPC-DCLs, selecting two of the most potent DCLs based on methylated β-CDs [7]. In the present study, the lipid bilayers of selected DCLs were additionally loaded with lipophilic mTHPC, achieving DL-DCLs. Physico-chemical characteristics (size, PDI, charge, and colloidal stability) of DL-DCLs were similar to those of single-loaded DCLs [7], and comparable with liposomal mTHPC formulation Foslip^®^ (Figure 2). According to the preparation technique, the ratio between aqueous volume of liposomes and void volume during the liposome preparation was low, explaining the low EE values of mTHPC in DL-DCLs [7,20]. The complete monomerization of mTHPC in all NPs was confirmed by absorption spectra (Figure 3a), and double encapsulation of mTHPC in DCLs was established using fluorescence spectroscopy (Figure 3b). The presence of mTHPC/CD complexes led to the increase of I_1_/I_2_ ratio [14], demonstrating I_1_/I_2_ = 0.88 and 1.00 for MD and TD, respectively, while in the case of Foslip^®^, I_1_/I_2_ was 0.8 (Figure 3b). At the same time, the high loading of mTHPC in the lipid bilayer resulted in fluorescence quenching [13,21], causing the decrease of FY, PIQ, and *r* values (Figure 3b, insert). Overall, 30% of mTHPC was encapsulated in CD complexes, while the fraction of PS in the lipid bilayer was 70%.

We further tested DL-DCLs in 2D and 3D in vitro tumor models. In 2D monolayer cell cultures, the behavior of both double-loaded mTHPC-DCLs was similar to that of Foslip^®^ (Figure 4 and Figure S1), while in 3D multicellular spheroids, we clearly demonstrated the benefits of matryoshka-type hybrid liposomes against conventional lipid vesicles. The 3D multicellular tumor spheroids more closely mimicked the native tumor environment, representing the tumor stroma tissue and offering better prediction potential when testing the penetration ability of nanomedicines [22]. The data of chemical extraction of mTHPC from spheroids demonstrated an almost similar amount of mTHPC at various incubation times (Figure 5a). However, detailed study using flow cytometry showed different profiles of mTHPC accumulation in the spheroid cells for DL-DCLs and Foslip^®^. The spheroids treated with TD displayed a homogeneous distribution between all cells at all time points for both HT29 (Figure 5d) and FaDu spheroids (Figure S2), while for MD and Foslip^®^, the accumulation of mTHPC was mainly associated with the PS uptake in a small fraction of cells (Figure 5b,c and Figure S2). At 24 h post-incubation, MD exhibited a slightly more homogeneous PS distribution in HT29 spheroids than Foslip^®^ (Figure 6). The fluorescence imaging data of spheroid frozen-cut sections confirmed the almost homogeneous distribution of mTHPC delivered by TD in both types of spheroid cells (Figure 6 and Figure S3). In the case of TD, the mTHPC fluorescence signal was uniform for the whole spheroid, while for MD and Foslip^®^, a strong peripheral fluorescence signal dropped to background values at 50 and 100 µM from the spheroid periphery, respectively. In all probability, the observed effect was related to the presence of mTHPC/CDs in the media due to DL-DCL disintegration, as has been previously observed for Foslip^®^ [10,23]. Indeed, in the case of TD, chromatography analysis of culture media after 24 h incubation of NPs with tumor spheroids demonstrated the presence of mTHPC/CD complexes. To confirm the serum-induced destruction of DL-DCLs, we demonstrated the increase of the mTHPC-TM-β-CD fraction in the medium supplemented with 9% of serum compared to 2% (Figure 7). It is worth noting that the MD-treated samples contained a similar amount of mTHPC in NPs, while all released mTHPC in the medium was localized only in serum proteins. Compared with TM-β-CD, Me-β-CDs possess less affinity to mTHPC [17]; thus, the equilibrium distribution of mTHPC is shifted to serum proteins [18]. Therefore, we suppose that the lifetime of mTHPC-Me-β-CDs was too short to penetrate the deep layers of spheroids, and mTHPC released from the MD was quickly redistributed to both serum proteins and the nearest tumor cells by Me-β-CDs, while long-living mTHPC-TM-β-CD complexes could easily penetrate to the deep tissue regions.

Finally, we assessed the PDT efficiency of TD and MD in 3D multicellular tumor spheroids in vitro, and of TD in xenografted mice in vivo. Despite the remarkable difference in penetration profiles, PDT-induced cell death in spheroids treated with TD and MD was similar to Foslip^®^. The same tendency was observed in the xenografted mice model: the difference in mean tumor growth delay for both TD and Foslip^®^ was about 30 days (Figure 8). We likely did not achieve the desired balance between the accumulation and distribution of PS in the tumor. We suggest that increasing the TD concentration could be helpful in exceeding the threshold of intracellular PS accumulation needed for efficient DCL-PDT photokilling. 

## 4. Materials and Methods 

### 4.1. Materials

mTHPC and its liposomal formulation (Foslip^®^) were kindly provided by biolitec research GmbH (Jena, Germany). The stock solution of mTHPC (2 mM) was prepared in methanol and kept at 4 °C in the dark. Foslip^®^, based on dipalmitoylphosphatidylcholine (DPPC) and dipalmitoylphosphatidylglycerol (DPPG) liposomes with a mTHPC/lipid ratio of 1:12 (mol/mol), was prepared by solubilizing powder in ultrapure water (UPW, Milli-Q^®^ Advantage A10^®^ System, Millipore, Eschborn, Germany) to a final mTHPC concentration of 2 mM. 

Random methyl-β-cyclodextrin (Me-β-CD; product code CY-2004.1,29; substitution degree of 12, average molecular weight 1135 Da) and heptakis(2,3,6-tri-O-methyl)-β-cyclodextrin (TM-β-CD; product code CY-2003,34; molecular weight 1429.6 Da) were purchased from CYCLOLAB R&D. Ltd., (Budapest, Hungary). DPPC and DPPG were purchased from Sigma (USA).

### 4.2. DL-DCL Preparation

DCLs were prepared by the thin lipid film hydration method, as described previously [7]. Briefly, inclusion complexes of mTHPC with β-CDs were formed using the solvent co-evaporation method in UPW. DPPC/DPPG liposomes loaded with mTHPC were prepared by membrane extrusion technique according to the previously published procedure, yielding unilamellar liposomes [24]. These liposomes contained DPPC and DPPG at a molar ratio of 9:1, with a final lipid concentration of 15 mg/mL. To obtain DL-DCLs, mTHPC was added at the step of the preparation of lipid mixture at a molar drug/lipid ratio of 1:15, and mTHPC/β-CD inclusion complexes were encapsulated at the lipid film hydration step. The purification of DCLs from the non-encapsulated mTHPC/β-CDs in the medium was performed using a minicolumn chromatography technique [25]. 

### 4.3. Cell Lines

HT29 human colon adenocarcinoma cell line was purchased from ATCC (LGC Promochem, Molsheim, France). FaDu (human pharynx squamous cell carcinoma) cell line was purchased from ATCC (Cat. No: ATCC1 HTB-43™). Cells were cultured in phenol red-free Roswell Park Memorial Institute 1640 medium (RPMI-1640, Invitrogen™, Carlsbad, California, USA), supplemented with 9% (vol/vol) heat-inactivated fetal bovine serum (Sigma-Aldrich, St. Louis, MO, USA), penicillin (10,000 IU) streptomycin (10,000 mg/mL) and 1% (vol/vol) 0.2 M glutamine (Invitrogen™, Carlsbad, California, USA). Cells were kept as a monolayer culture in a humidified incubator (5% CO_2_) at 37 °C. Cell culture was reseeded every week to ensure exponential growth. 

### 4.4. Multicellular Tumor Spheroid Model

HT29 MCTSs were initiated as previously described [26]. Briefly, flasks coated with 1% L-agarose were seeded with 5 × 10^4^ HT29 cells/mL. After three days, cellular aggregates were transferred into spinner flasks (Integra Biosciences, Cergy Pontoise, France) containing 125 mL RPMI-1640 medium supplemented with 9% FBS. Spinner flasks were placed under constant agitation at 75 rpm at 37 °C (5% CO_2_, humidified atmosphere) for 15 days. Spheroids were filtered to approximatively 500 µm in diameter before conducting experiments. 

MCTSs were generated from FaDu cells using the liquid overlay technique (LOT), as described previously [27]. Briefly, 100 µL of FaDu cells (5 × 10^4^ cells/ml) and 100 µL of full RPMI medium were added to each well of a 96-well plate previously coated with 1% agarose (w/v in water), and cultured at 37 °C, 5% CO_2_ for 5 days before being taken into experiments.

For dissociation, spheroids were transferred into a 12-well plate, washed twice with PBS and further incubated with 0.025% trypsin (GIBCO™, ThermoFisher, Waltham, MA, USA) and 0.01% ethylenediaminetetraacetic acid (GIBCO™, ThermoFisher, Waltham, MA, USA). For complete trypsinization, the plate with spheroids was placed on a rotatory shaker (60 rpm) for 30 min in subdued light. After dissociation, trypsin action was inhibited by the addition of 3 mL complete culture medium (9% FBS), and the cell suspension was centrifuged to a pellet and further re-suspended in a fresh medium.

### 4.5. Fluorescence Staining

For in vitro cell experiments, stock NP solutions were diluted in RPMI-1640 supplemented with 2% heat-inactivated fetal bovine serum (FBS, Life Technologies, Carlsbad, California, USA) to obtain the final mTHPC concentration of 1.5 µM for 2D monolayer cells and 4.5 µM for 3D tumor spheroid experimentation.

For the 2D monolayer cell culture, cells (5 × 10^4^ cells/mL) were seeded in 24-well plates for 72 h and then incubated with Foslip^®^, MD, or TD (1.5 µM). In the case of 3D cell culture, before incubation with mTHPC NPs (4.5 µM), spheroids were washed with serum-free RPMI-1640 medium. PS incubation was performed in the dark at 37 °C in a humidified 5% CO_2_ air atmosphere.

### 4.6. Animal Model

All experiments were performed in accordance with animal care guidelines from the European Union and were approved by the appropriate authority. The animal project registered under the number (#2438) received a favorable assessment from the Ethics Committee and was approved by the French Higher Education and Research Minister. All procedures involving animals were performed under general anesthesia with inhaled isoflurane (Vetflurane; Virbac, France) using a Univentor 400 anesthesia unit (Genestil, Royaucourt, France). Mice were housed in filtered air cabinets with a 12 h light/dark cycle at 22–24 °C and 50% humidity, provided with food and water ad libitum, and manipulated following aseptic procedures. Female NMRInu/nu mice (Janvier, St Berthevin, France) aged 9–10 weeks were used, with a mean bodyweight of 30 ± 3 g. Mice were inoculated subcutaneously in the left flank with 8 × 10^6^ exponentially growing HT29 cells and the experiments were initiated 5–7 days after inoculation when the tumors reached 50 mm^3^ in volume. 

### 4.7. Analytical Techniques

#### 4.7.1. Spectroscopy

Absorption measurements were recorded with a Lambda 35 spectrometer (Perkin Elmer, USA) and fluorescence measurements were conducted with a LS55B spectrofluorometer (PerkinElmer, USA) equipped with polarizers, thermostated cuvette compartments, and magnetic stirring for polarization experiments. The concentrations of mTHPC in DL-DCLs and Foslip^®^ were estimated spectroscopically (λem = 652 nm) by dissolving nanoparticles in methanol. DL-DCLs were previously purified by minicolumn chromatography. Fluorescence quantum yield and photoinduced fluorescence quenching were measured as previously described (λ_ex_: 416 nm; λ_em_: 652 nm) [15]. The measurements of mTHPC fluorescence anisotropy were performed as described earlier (λ_ex_: 430 nm; λ_em_: 652 nm) [15]. EE of mTHPC in DCLs was measured spectroscopically (λ_em_: 652 nm) immediately after extrusion and purification, as previously described [7]. 

The hydrodynamic diameter of NPs, polydispersity index, and Z-potential were determined using photon-correlated spectroscopy by a Zetasizer Nano ZS (Malvern Instruments, UK) as previously reported [7].

#### 4.7.2. Flow Cytometry

Flow cytometry analysis was performed using a FACSCalibur (BD, Franklin Lakes, NJ, USA), equipped with lasers emitting at 488 nm and 633 nm. Flow cytometry histograms were obtained from the suspension of cells after dissociation of 50 NP-treated spheroids. The fluorescence of mTHPC was detected in the fluorescence channel FL4 with a 661 ± 16 nm filter under excitation at 633 nm. Propidium iodide (PI) fluorescence was detected in the FL2 channel with a 585 ± 42 nm filter (excitation at 488 nm). Data analysis was carried out using Flowing Software (Turku Centre for Biotechnology, Turku, Finland).

#### 4.7.3. Fluorescence Microscopy

HT29 cells (3 × 10^4^ cells/mL) were plated into Lab-Tek II chamber Slide (Roskilde, Denmark), incubated in the dark at 37 °C with 1.5 μM of mTHPC in different formulations for 3 h and then rinsed with PBS. mTHPC fluorescence was observed with a confocal laser-scanning microscope (Leica SP5 X AOBS LCSM, Leica microsystem, Wetzlar, Germany). For the 2D monolayer cell culture, fluorescence images were recorded using an oil immersion ×40 objective. The 3D tumor spheroids were embedded into a resin Shandon^TM^ Cryomatrix^TM^ (ThermoFisher, Waltham, MA, USA), frozen, cut, and 20 µm thick cryosections were further analyzed by confocal microscopy (×10 objective). Fluorescence of mTHPC in FaDu 2D monolayer cells and frozen-cut cryosections of FaDu spheroids was analyzed using an upright epifluorescence microscope (AX-70 Provis, Olympus, France). The fluorescence images were obtained using a filter set at 405–445 nm excitation associated with a 570 nm dichroic mirror and a 590 nm long-pass emission filter for fluorescence measurements. 

Analysis of the images was performed with ImageJ (NIH, USA) software. To estimate the penetration profile of dye in the spheroids, special macros were proposed. Briefly, the spheroid area was divided into 100 concentric rims, with the diameter decreasing in a linear way. After that we calculated the mean intensity of pixels in each rim. The final profiles were plotted as mean ± standard deviation from different cryo-sections (*n* = 7).

#### 4.7.4. Uptake in Spheroids 

mTHPC uptake in spheroids was measured by chemical extraction of mTHPC. After 3, 6, and 24 h incubation with NPs (4.5 µM), spheroids were dissociated and individual cells were subjected to the extraction of mTHPC with ethanol (99.6%) as previously described [11]. Briefly, after sonication (15 min) and centrifugation (5 min, 1500 rpm), mTHPC fluorescence in the supernatant was assessed (λ_ex_: 420 nm; λ_em_: 652 nm). 

#### 4.7.5. Chromatography

Chromatographic experiments were performed on a Sigma 1.5 × 75 cm column filled with Sepharose CL-6B gel (GE Healthcare, USA) pre-equilibrated with PBS, with a total bed volume of 150 mL. HT29 tumor spheroids were incubated in RPMI-1640 media which was supplemented with 2% and 9% FBS. The NPs were added at final mTHPC concentration of 4.5 µM. After 24 h incubation, the supernatant was centrifuged (5 min, 1500 rpm), filtered using a 450 µm Millex^®^ – HV syringe driven filter unit (Sigma-Aldrich, St. Louis, MO, USA), and injected into the column using a three-way connector. Fractions of 1 mL were collected by an automated fraction collector. The column was stored at room temperature and separation was carried out in a partially light-protected environment to avoid mTHPC photobleaching. Fractions with elution volumes from 25 to 120 mL were collected and analyzed for mTHPC content using a SAFAS Xenius XM (SAFAS, Monaco, France) spectrofluorometer, as previosly reported [28]. The mTHPC content in the chromatographic fractions was estimated by measurements the fluorescence intensity after the addition of 0.2% Triton^®^ X-100 to the samples. The analysis of chromatography histograms was performed using Origin software (OriginLab, Northampton, MA, USA).

### 4.8. Photoirradiation of Spheroids

HT29 spheroids were transferred from the spinner flask to 12-well plates and incubated with NPs for 24 h (4.5 µM). Spheroids were then washed and subjected to irradiation. Irradiation was performed at 652 nm with a Ceralas PDT diode laser (CeramOptec GmbH, Bonn, Germany) at 40 J/cm^2^ (fluence rate of 90 mW/cm^2^). Control spheroids were exposed to mTHPC only (drug, no light). The viability of the spheroid cells was assessed by propidium iodide probe. Spheroids were trypsinated 6 h post-PDT and the obtained cell suspension was stained with 1 µg/mL PI (Biolegend, San Diego, CA, USA) for 15 min in the dark at 37 °C, rinsed, and analyzed by flow cytometry.

### 4.9. Animal Experiments

NPs were administered intravenously by a tail vein injection at a dose of 0.15 mg/kg of mTHPC. Following the injection, mice were kept in the dark, and experiments were undertaken with minimal ambient light. Tumor irradiation was performed at 652 nm with a Ceralas PDT diode laser. The mice were treated 24 h post-administration at a fluence of 10 J/cm^2^ and the fluence rate of 100 mW/cm^2^. Just after PDT and 24 h later, mice received analgesia by subcutaneous injection of a mixture of 0.08 mg/kg buprenorphine (Axience, Pantin, France) and 1 mg/kg of the non-steroid anti-inflammatory Metacam (Boehringer Ingelhein, Ingelheim, Germany) Mice were kept in the dark for 7 days after PDT. Three times per week, the perpendicular diameters of the tumors were measured to document tumor growth. Tumor volume (V) was calculated using the equation: V = W^2^ × Y/2, where (W) and (Y) are the smaller and larger diameters. Mice were sacrificed when tumor volume reached the ethical size of 1000 mm^3^.

### 4.10. Statistics

The data from at least three independent experiments are presented as mean ± standard deviation. The data were evaluated using nonparametric Mann–Whitney U test (StatView^TM^ software) with a significance level of *p* < 0.05.

## 5. Conclusions

The complexity of drug distribution processes requires innovative approaches in designing drug delivery platforms. Hybrid delivery systems have proven to be a powerful tool capable of combining the benefits of individual NPs intp one nanoplatform. Our study confirmed the advantage of double-loaded mTHPC-DCLs over a conventional mTHPC liposomal formulation (Foslip^®^) in terms of tumor tissue penetration. Altogether, the proposed matryoshka-type lipid vesicles releasing mTHPC/CD complexes illustrated an optimal PS distribution in in vitro 3D models of tumor tissue.

Unexpectedly, we did not find phototoxic benefits of matryoshka-type liposomes over Foslip^®^ in multicellular spheroids in vitro or in an animal experiment in vivo, at least under our experimental conditions. Our ongoing studies address different experimental settings, aiming for matryoshka system optimization in preclinical models. One of the possible solutions is the incorporation of stimulus-response moieties in liposomes to promote the release of CD nanoshuttles at the target site. 

## Supplementary Materials

The following are available online at https://www.mdpi.com/2072-6694/11/9/1366/s1, Figure S1: Typical epifluorescence images of mTHPC fluorescence in FaDu monolayer cells at 3 h post-incubation with Foslip^®^, MD, and TD; Figure S2: Flow cytometry histograms of HT29 spheroids treated with Foslip^®^, MD, and TD for 24 h. Figure S3: Typical flow cytometry histograms of FaDu spheroids treated with Foslip^®^, MD, and TD for 24 h; Figure S4: Typical brightfield/fluorescence overlay images of mTHPC in FaDu spheroid cryosections after 24 h incubation with various mTHPC formulations (Foslip^®^, MD, and TD).
