# Supplementary material for: Matryoshka-Type Liposomes Offer the Improved Delivery of Temoporfin to Tumor Spheroids
Source: Cancers (Basel). 2019 Sep 13;11(9):1366. doi: 10.3390/cancers11091366 (PMC6770699; doi:10.3390/cancers11091366)
Supplement: Supplementary file 1 [file cancers-11-01366-s001.pdf]

Supplementary material: Matryoshka-type liposomes offer the improved delivery of temoporfin to tumor spheroids

Ilya Yakavets, Marie Millard, Laureline Lamy, Aurelie Francois, Dietrich Scheglmann, Arno Wiehe, Henri-Pierre Lassalle, Vladimir Zorin, Lina Bezdetnaya

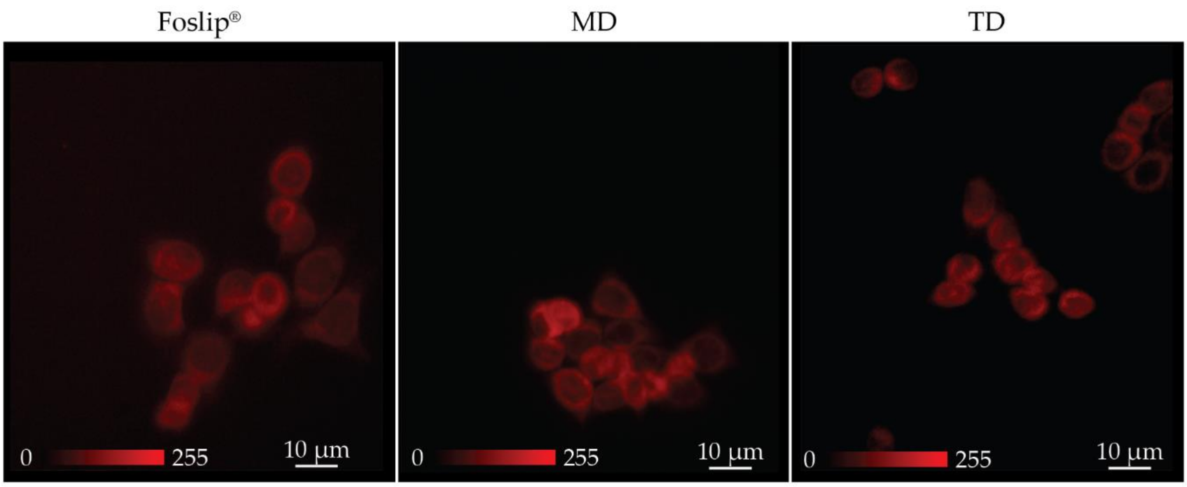

Figure S1. Typical epifluorescence images of mTHPC fluorescence in FaDu monolayer cells at 3h post-incubation with Foslip®, MD and TD. Scale bar: 10 μm. The concentration of mTHPC was 1.5 μM. Serum concentration was 2 %.

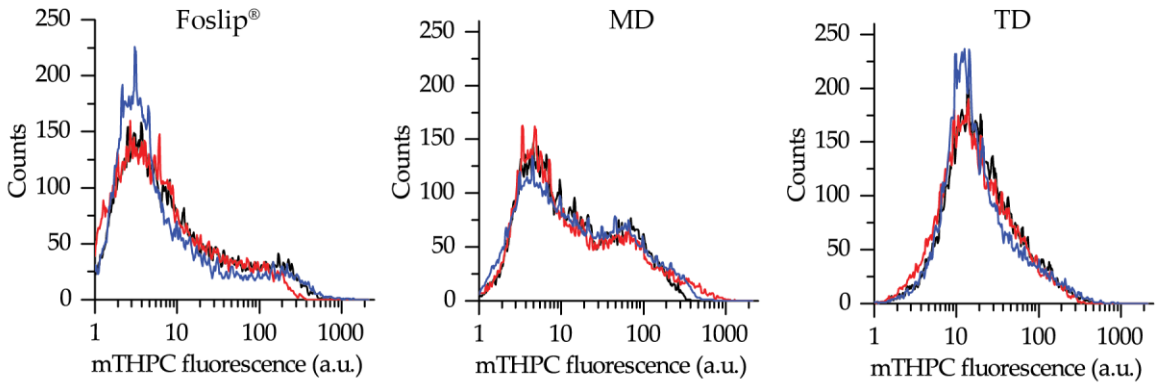

Figure S2. Flow cytometry histograms of HT29 spheroids treated with Foslip®, MD and TD for 24h. Each plot represents 3 independent experiments. mTHPC concentration was 4.5 μM. Serum concentration was 2 %.

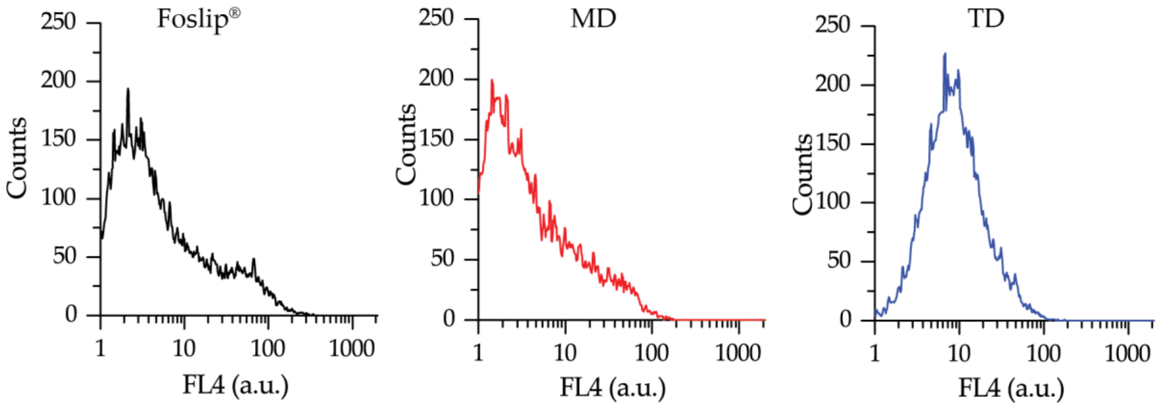

Figure S3. Typical flow cytometry histograms of FaDu spheroids treated with Foslip®, MD and TD for 24h. mTHPC concentration was 4.5 µM. Serum concentration was 2 %.

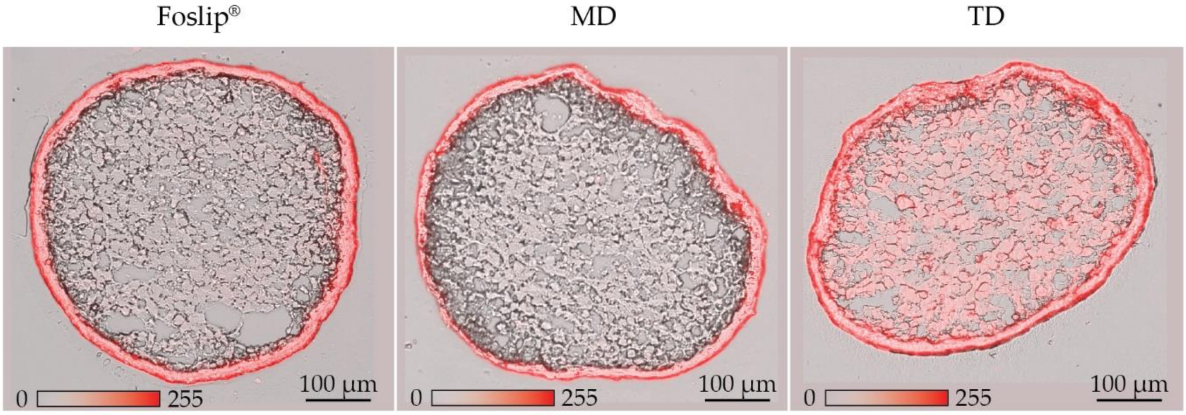

Figure S4. Typical brightfield/fluorescence overlay images of mTHPC in FaDu spheroids cryosections after 24h incubation with various mTHPC formulations (Foslip®, MD and TD). mTHPC concentration was 4.5 µM. Serum concentration was 2 %. Fluorescence of mTHPC is displayed in red-color.

© 2019 by the authors. Licensee MDPI, Basel, Switzerland. This article is an open access article distributed under the terms and conditions of the Creative Commons Attribution (CC BY) license (http://creativecommons.org/licenses/by/4.0/).
